# Enhanced neuronal activity by suffruticosol A extracted from *Paeonia lactiflora* via partly BDNF signaling in scopolamine-induced memory-impaired mice

**DOI:** 10.1038/s41598-023-38773-8

**Published:** 2023-07-20

**Authors:** June Hee Kim, Young-Eun Han, Soo-Jin Oh, Bonggi Lee, Obin Kwon, Chun Whan Choi, Min Soo Kim

**Affiliations:** 1 Brain Science Institute, Korea Institute of Science and Technology (KIST), Seoul, 02792 Republic of Korea; 2 Division of Bio-Medical Science and Technology, KIST School, University of Science and Technology (UST), Seoul, 02792 Republic of Korea; 3 Department of Food Science and Nutrition, Pukyong National University, Busan, 48513 Republic of Korea; 4 Department of Biomedical Sciences, Seoul National University College of Medicine, Seoul, 03080 Republic of Korea; 5 Natural Biomaterial Team, Biocenter, Gyeonggido Business and Science Accelerator, Suwon, 16229 Gyeonggi-do Republic of Korea

**Keywords:** Drug discovery, Molecular biology, Neuroscience, Health care, Molecular medicine

## Abstract

Neurodegenerative diseases are explained by progressive defects of cognitive function and memory. These defects of cognition and memory dysfunction can be induced by the loss of brain-derived neurotrophic factors (BDNF) signaling. *Paeonia lactiflora* is a traditionally used medicinal herb in Asian countries and some beneficial effects have been reported, including anti-oxidative, anti-inflammatory, anti-cancer activity, and potential neuroprotective effects recently. In this study, we found that suffruticosol A is a major compound in seeds of *Paeonia lactiflora*. When treated in a SH-SY5 cell line for measuring cell viability and cell survival, suffruticosol A increased cell viability (at 20 µM) and recovered scopolamine-induced neurodegenerative characteristics in the cells. To further confirm its neural amelioration effects in the animals, suffruticosol A (4 or 15 ng, twice a week) was administered into the third ventricle beside the brain of C57BL/6 mice for one month then the scopolamine was intraperitoneally injected into these mice to induce impairments of cognition and memory before conducting behavioral experiments. Central administration of suffruticosol A into the brain restored the memory and cognition behaviors in mice that received the scopolamine. Consistently, the central treatments of suffruticosol A showed rescued cholinergic deficits and BDNF signaling in the hippocampus of mice. Finally, we measured the long-term potentiation (LTP) in the hippocampal CA3–CA1 synapse to figure out the restoration of the synaptic mechanism of learning and memory. Bath application of suffruticosol A (40 µM) improved LTP impairment induced by scopolamine in hippocampal slices. In conclusion, the central administration of suffruticosol A ameliorated neuronal effects partly through elevated BDNF signaling.

## Introduction

Alzheimer's disease (AD), the most prevalent type of dementia, is characterized by a progressive deterioration of cognitive and memory functions^1,2^. This loss of functions in AD patients is associated with the disruption of hippocampal cholinergic neurons^3,4^. The cholinergic system has a crucial role in memory and cognition processes in the central nervous system^3,5^. Depletion of cholinergic response in AD is correlated with β-amyloid (Aβ) plaques and tau neurofibrillary tangles. Additionally, further evidence suggests that the cholinergic deficit worsens Aβ and exacerbates tau pathology in an animal model^5^. To improve cholinergic levels, acetylcholinesterase (AChE) inhibitors, such as donepezil, rivastigmine, and galantamine, are administered to AD patients. Supplementing these drugs for a duration of 6 months or longer has been shown to enhance acetylcholine (ACh) activity and delay the progression of AD-related symptoms^6,7^. However, these drugs are only approved for mild to moderate dementia symptoms and are not recommended for patients with mild cognitive impairment^7^. Moreover, patients taking these drugs have reported gastrointestinal side effects attributed to their chemical properties^8^. These limitations underscore the need to discover a novel compound for AChE inhibition.

A mouse model of scopolamine-induced cognitive impairment is widely used for AD drug investigation. This model damages cholinergic system, which interrupt memory formation which is similar as AD patient^9^. Coincide downregulations of brain-derived neurotrophic factor (BDNF) and its regulator, cAMP-response element-binding protein (CREB) appear in scopolamine treated hippocampus^10^. BDNF and CREB are involved in modulating synaptic plasticity and memory formation^11,12^. Previous studies have reported decreased serum levels and hippocampal expression of BDNF in AD patients^13,14^. Restoring BDNF levels has been shown to rescue neuronal loss, enhance cholinergic activities in the hippocampus, and improve spatial learning in animal models^15–17^. Thus, BDNF signaling pathways have been explored as potential targets for developing novel therapeutic agents for AD treatment.

*Paeonia lactiflora* is a medicinal herb traditionally used in some Asian countries^18^. Recent reports have revealed that *P. lactiflora* exhibits anti-oxidative, anti-inflammatory, and anti-cancer activity^18–22^, and it has also been shown to rescue neural activities^23^. *P. lactiflora* seeds contain several resveratrol oligomers. Resveratrol, a well-studied natural monomeric stilbene, has a wide range of therapeutic benefits including anti-inflammation, cardio-protection, cancer prevention, and neural amelioration^24,25^. However, resveratrol has limitations in terms of therapeutic effect due to its poor bioavailability and debates regarding its biosafety. Recent studies have reported enhanced therapeutic efficacy of resveratrol oligomers^26^. Therefore, applying resveratrol oligomers to AD patients can be a potent treatment for AD. One of the resveratrol oligomers found in *P. lactiflora* seeds is suffruticosol A^27^. An in vitro study reported that suffruticosol A can inhibit beta-site APP cleaving enzyme 1 (BACE-1)^27^, which can decrease brain Aβ level^28^. Suffruticosol A has also been shown to have an anti-inflammatory effect in vitro and in vivo in pro-inflammatory conditions^29^. Furthermore, some stilbene derivatives have demonstrated AChE-inhibition activity^30–32^. Previous studies have suggested that suffruticosol A can be a potent neuroprotectant, although further studies are necessary. In this study, we focused on the neuroprotective effect of suffruticosol A both in vitro and in vivo. We used a scopolamine-treated SH-SY5Y cell line to demonstrate the ameliorating effect of suffruticosol A against scopolamine in vitro. Additionally, we investigated the potential restoration of suffruticosol A in a scopolamine-induced memory impairment model. The underlying mechanism of suffruticosol A on the scopolamine model was examined at the molecular level by evaluating cholinergic activity and BDNF signaling pathways in the hippocampus.

## Materials and methods

### Isolation of resveratrol oligomer from *Paeonia lactiflora* seed

#### Plant material

The seeds of *P. lactiflora* were harvested from the herb garden of the Medicinal Plant Experiment Station, located in Uisong (Korea), and were identified by one of the authors (Dr. C. W. Choi, GBSA, Suwon). The collection of plant seeds complied with relevant institutional, national, and international guidelines and legislation. A voucher specimen (KR1072) was estimated in the Korea Research Institute of Chemical Technology (KRICT) and deposited at its herbarium.

#### Extraction and isolation

**Figure 1:**
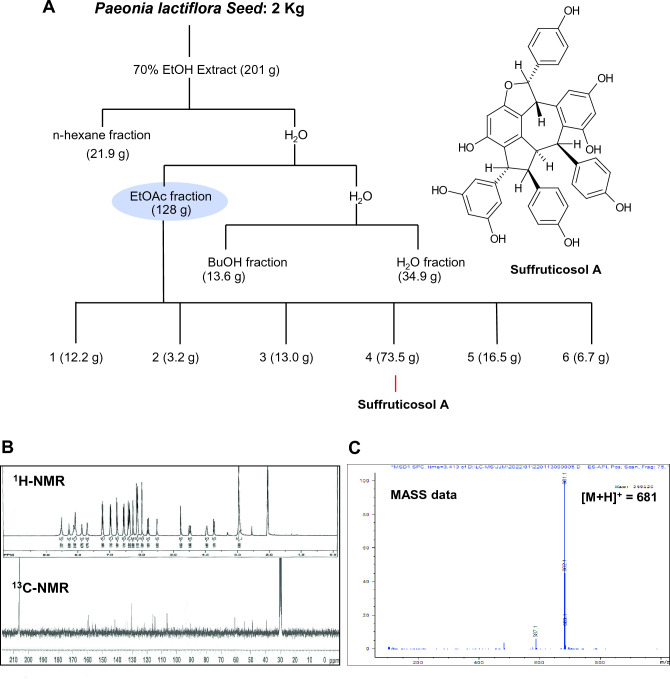
Isolation and structural analysis of suffruticosol A isolated from seeds of *P. lactiflora.* (**A**) Extraction and purification procedures of suffruticosol A of seeds of *P. lactiflora* and its molecular structure. (**B**) ^1^H-NMR and ^13^C-NMR (300 MHz, acedone-*d*6) spectrums of suffruticosol A of seeds of *P. lactiflora.* (**C**) Mass spectrum of suffruticosol A of seeds of *P. lactiflora.*

Dried seeds of *P. lactiflora* (2 kg) were extracted with 10 L of mixed (70% EtOH) and then evaporated to dryness, resulting in 201 g of dark syrupy extract. This extract was suspended in H_2_O (5 L) and separated consequently with an equal volume of ethyl acetate (EtOAc), *n*-butanol (*n*-BuOH), and *n*-hexane, yielding an EtOAc soluble fraction (128.6 g), a *n*-BuOH soluble fraction (13.6 g), a *n*-hexane soluble fraction (21.9 g) and a residual aqueous fraction (34.9 g). The EtOAc soluble fraction (128.6 g) was placed in silica gel (1.5 kg) column (Ø = 8.0 × 60 cm) chromatography, eluted with MeOH in CH_2_Cl_2_ according to a step-gradient manner (1% to 50%) to give six fractions (F1: 12.2 g, F2: 3.2 g, F3: 13.0 g, F4: 73.5 g, F5: 16.5 g and F6: 6.7 g). Finally, we achieved the purification of suffruticosol A (31 g) from repeated RP-18 chromatography of F4 with step-gradient elution of MeOH in H_2_O (Fig. 1A).

### Spectroscopy of isolated suffruticosol A from *Paeonia lactiflora* seed

Compound; yellow amorphous powder; ^1^H-NMR (300 MHz, CD_3_OD) δ: 7.10 (2H, d, *J* = 8.8 Hz, H-2'', 6''), 6.95 (2H, d, *J* = 8.8 Hz, H-2, 6), 6.68 (2H, d, *J* = 8.8 Hz, H-3'', 5''), , 6.37 (2H, d, *J* = 8.8 Hz, H-3, 5), 6.48 (2H, d, *J* = 8.8 Hz, 2', 6'), 6.25 (1H, d, *J* = 2.2 Hz, H-12''), 6.12 (2H, d, *J* = 8.8 Hz, H-3', 5'), 6.19 (1H, d, *J* = 0.8 Hz, H-12'), 6.06 (1H, t, *J* = 2.2 Hz, H-12), 5.98 (2H, d, *J* = 2.2 Hz, H-10, 14), 5.68 (1H, d, *J* = 11.2 Hz, H-7''), 5.93 (1H, d, *J* = 1.8 Hz, H-14''), 4.23 (1H, d, *J* = 3.2 Hz, H-7'), 4.74 (1H, s, H-8), 4.35 (1H, d, *J* = 11.2 Hz, H-8''), 3.68 (1H, d, *J* = 8.0 Hz, H-7), 3.93 (1H, m, H-8'). ^13^C-NMR (75 MHz, CD_3_OD) δ: 160.2 (C-11'), 159.3 (C-11, 13), 158.9 (C-4''), 156.7 (C-13''), 156.5 (C-4), 155.1 (C-13'), 154.9 (C-11''), 154.5 (C-4'), 148.4 (C-9), 144.7 (C-9'), 141.8 (C-9''), 135.5 (C-1), 133.9 (C-1'), 130.8 (C-1''), 130.7 (C-2', 6'), 130.7 (C-2, 6), 130.5 (C-2'', 6''), 126.9 (C-10''), 123.0 (C-14'), 117.3 (C-10'), 116.2 (C-3'', 5''), 115.4 (C-3, 5), 114.2 (C-3', 5'), 106.8 (C-10, 14), 105.9 (C-14''), 101.9 (C-12''), 101.4 (C-12), 96.2 (C-12'), 91.5 (C-7''), 61.0 (C-7), 54.6 (C-8), 48.8 (C-8''), 48.6 (C-8'), 39.8 (C-7').

### Cell culture and treatment

The human neuroblastoma cell line, SH-SY5Y cells were purchased from ATCC. These cells were cultured under the following media condition: High glucose Dulbecco's modified Eagle's medium (DMEM; HyClone, S, Logan, UT, USA) supplemented with 10% fetal bovine serum (Gibco, Grand Island, NY, USA) and penicillin–streptomycin (10,000 U/mL; Grand Island, NY, Gibco). The cells were incubated in a condition of 5% CO_2_ humidified atmosphere at 37 °C. Cell subcultures were performed every 3–4 days using trypsin–EDTA (Sigma, Saint Louis, MO, USA) for cell detachment. For the experiments, 2 × 10^4^ cells were seeded in a 96-well plate in 100 μL of culture medium and pre-incubated overnight to allow cell attachment. The next day, suffruticosol A (1, 5, or 20 μM) and scopolamine hydrobromide trihydrate (0 or 2 mM; Sigma, Saint Louis, MO, USA) was added to a final volume of 200 μL medium, followed by further incubation of 24 h.

### Cell viability experiment

Following the treatment, cell viability, and cell survival effects against scopolamine were assessed by MTT Assay Kit (Abcam, Cambridge, UK) according to the manufacturer’s instructions. Briefly, the cell media and treatment were gently discarded using a micropipette to minimize any potential color interference of suffruticosol A. Next, 50 μL of serum-free medium and 50 μL of MTT reagent were added to each well. After applying the MTT solution, the cells were incubated for 3 h, followed by the addition of 150 μL of MTT solvent to each well. The plate was wrapped in foil to prevent light exposure and shaken using an orbital shaker. The absorbance was measured at 590 nm using a Tecan Infinite 200 microplate reader (Tecan, Männedorf, Switzerland). Cell viability and neuroprotection effects were expressed as a percentage relative to the control group.

### Animals

C57BL/6 male mice without any genetic modification (8 weeks old; 20–28 g) were obtained from DBL Inc. (Eumseong-gun, Korea). The mice were housed in a controlled environment with a 12–hour light/12–hour dark cycle, a temperature of 23 ± 1 °C, a humidity of 50 ± 10%, and had free access to water and food. The mice were acclimated for one week before the experiments. All experimental procedures involving animals were approved by the Institutional Animal Care and Use Committee (IACUC) and the Institutional Biosafety Committee (IBC) at the Korea Institute of Science and Technology (KIST) (Approval number KIST-2021-04-046). The experimental procedures followed the ARRIVE guidelines and adhered to the American Veterinary Medical Association (AVMA) Guidelines for the Euthanasia of Animals (2020).

### Cannulation procedure and treatments

The mice underwent cannula implantation into the hypothalamic third ventricle (3 V) using a modified version of the procedure described previously^33^. A 26-gauge guide cannula (Plastics One, Roanoke, VA, USA) was implanted using an ultraprecise small animal stereotactic apparatus (Kopf Instruments, Tujunga, CA, USA). The coordinates for cannula implantation were 1.5 mm posterior to the bregma and 5.0 mm below the bregma. All mice underwent cannulation regardless of the treatment. Following the surgery, the animals had a one-week recovery period. The animals were randomly divided into the following groups (n = 5/group): Control (Con, treated with phosphate-buffered saline (PBS) as a vehicle), scopolamine + vehicle (Scop + Veh, treated with PBS as a vehicle), scopolamine + 4 ng suffruticosol A (Scop + Suff/L), and scopolamine + 15 ng suffruticosol A (Scop + Suff/H). All groups received injections of either the vehicle (0.5 μL PBS) or suffruticosol A (4 ng or 15 ng suffruticosol A in 0.5 μL PBS) into the third ventricle of the brain twice a week using the cannula. Scopolamine (1.0 mg/kg, dissolved in 0.9% saline) was injected intraperitoneally (i.p.) 30 min before all behavioral tests.

### Behavioral tests

The following behavioral tests were conducted in the behavioral testing room. For the open field test and Y-maze test, the movement of all mice was tracked and analyzed using the Anymaze video-tracking system (Stoelting, Wood Dale, IL, USA) and a computer equipped with a digital camera. The experiments were conducted in compliance with the ARRIVE guidelines. Open Field Test: The open field test was performed as previously described^34^. A white chamber measuring 40 cm in length, 40 cm in width, and 50 cm in height was used for the open field test. The mouse was placed in the center of the chamber and allowed to acclimate for 20 min. After 24 h of acclimation, all mice were placed in the same chamber for tracking and the total distance moved (in meters) was measured. Y-maze test: the Y-maze test was performed as previously described^35^. The Y-maze consisted of three identical arms measuring 40 cm in length, 4 cm in width, and 15 cm in height, arranged at 120° angles. Visual cues were placed at the end of each arm. The mouse was gently placed at the end of one arm with its head facing away from the center. The mouse was then allowed to freely explore all three arms for 10 min. During the exploration, the number of entries into each arm was recorded. Spontaneous alternation was measured as sequential entries into all three arms (e.g., ABC, BCA, CAB, but not ABA), excluding repeated entries into the same arm. The percentage of spontaneous alternation was calculated using the following formula: Percentage = [(Number of spontaneous alternations)/(Total arm entries − 2)] × 100. Passive Avoidance Test: the passive avoidance test was performed as described previously^36^. The test was conducted using an Avoidance System (B.S. Technolab INC., Seoul, Korea). The apparatus consisted of two chambers, a light chamber, and a dark chamber, separated by a gate in the middle. For acclimation, each mouse was placed in the light chamber with the gate open and allowed to freely explore both chambers for 10 min. After 24 h, a training session was conducted. The mouse was gently placed in the light chamber with the door closed, and after 60 s, the gate was opened. The step-through latency, or the time taken for the mouse to enter the dark chamber, was recorded. Once the mouse entered the dark chamber, the gate was closed and a mild electrical shock (0.3 mA, 3 s) was applied to the mouse's foot. On the following day (probe trial), the mouse was placed in the light chamber with the door closed, and after 60 s, the gates were opened. The step-through latency was recorded for up to 300 s.

### Electrophysiology

Adult mice (C57/BL6J, age 5–6 weeks) were anesthetized using isoflurane. The brain was rapidly removed and immersed in an ice-cold oxygenated sucrose-based dissection buffer containing the following concentrations (in mM): 1.23 NaH_2_PO_4_, 5 KCl, 0.5 CaCl_2_, 26 NaHCO_3_, 10 MgSO_4,_ and 212.5 sucrose saturated with 5% CO_2_–95% O_2_, at pH 7.4. The brain was then mounted on the stage of a vibrating microtome (DSK Linear Slicer NLS-MT, Kyoto, Japan), and transverse slices of 400 µm thickness were sectioned. The slices were then transferred to an incubation chamber at room temperature and allowed to recover for one hour before recording, in standard oxygenated artificial cerebrospinal fluid (aCSF) composed of the following concentrations (in mM): 24 NaHCO_3_, 130 NaCl, 1.25 NaH_2_PO_4_, 3.5 KCl, 1.5 MgCl_2_, 1.5 CaCl_2_, and 10 glucose saturated with 95% O_2_–5% CO_2_, at pH 7.4. Slices of the hippocampus were mounted on the stage and superfused with oxygenated aCSF at 28 °C. A concentric bipolar electrode (CBBPE75, FHC, Bowdoin, ME, USA) stimulated the Schaffer collateral pathway, and we recorded field excitatory postsynaptic potential (fEPSP) from the stratum radiatum of CA1 using a glass pipette filled with aCSF (1–3 MΩ). The evoked fEPSP responses were intensified by a Multiclamp 700b (Axon Instruments, San Jose, CA, USA) and expressed by Digidata 1322A. The slope of the fEPSP response was calculated by using pCLAMP 10 software (Axon Instruments, San Jose, CA, USA). The stimulation intensity was attuned to obtain fEPSP slopes of 50–60% to the maximum. Bath temperature was maintained at 28 °C by a temperature controller (TC344B, Warner Instrument Corporation, Hamden, CT, USA) during recordings. The basal slope of the fEPSP was monitored by electrical stimulation at 0.1 Hz. For the long-term potentiation (LTP) induction, electrical stimulations were applied as theta-burst stimulation (TBS), consisting of three trains of stimuli. Each train is composed of five burst stimuli transported at 5 Hz (every 200 ms), with each burst comprising four pulses at 100 Hz.

### Acetylcholine activity assays

Choline acetyltransferase level assay: the hippocampus was homogenized in PBS on ice. For the collection of hippocampal supernatants, homogenized solutions were centrifuged at 1,500×*g* for 15 min. The supernatants were used to analyze choline acetyltransferase (ChAT) levels with a ChAT ELISA kit (MyBioSource, MBS724080, San Diego, CA, USA) according to the manufacturer’s instructions. The ChAT activity was measured by the absorbance at 450 nm. The enzyme activity was calculated as follows: Enzyme activity (unit/mg protein) = [(∆A324)/ × 16.6]/(1.98 × 10^−5^ nM^−1^ cm^−1^ × 24)/[protein concentration (mg/mL)]. To normalize enzyme activity, protein concentrations were assayed using the Quick Start Bradford Protein Assay kit (Bio-Rad, Hercules, CA, USA). Acetylcholine and acetylcholineesterase assay: The mouse hippocampus was homogenized on ice in RIPA buffer (Merck KGaA, Darmstadt, Germany) with PhosSTOP (Roche, Mannheim, Germany) and cOmplete Mini EDTA-free Protease Inhibitor Cocktail (Roche, Mannheim, Germany) supplemented. Centrifugation for the homogenates was conducted at 16,000×*g* for 20 min. The supernatants were collected to evaluate acetylcholine (ACh) level and acetylcholinesterase (AChE) activity. Amplex™ Acetylcholine/Acetylcholinesterase Assay Kit (Invitrogen, Waltham, MA, USA) was used to measure ACh and AChE activity according to the manufacturer’s protocol. ACh and AChE levels were measured with wavelengths of 563 nm and calculated from a standard curve.

### The mRNA isolation, cDNA synthesis, and quantitative real-time PCR

Hippocampal mRNA extraction was performed using TRIzol reagent (Invitrogen, Waltham, MA, USA). The hippocampal mRNA was synthesized to complementary DNA (cDNA) using the SuperScript III First-Strand Synthesis System for RT-PCR (Invitrogen, Waltham, MA, USA) according to the manufacturer’s protocols. The Power SYBR Green PCR Master Mix kit (Applied Biosystems, Waltham, MA, USA) was utilized for the cDNA amplification. The QuantStudio 3 Real-Time PCR Instrument (Applied Biosystems, Waltham, MA, USA) was used for quantitative real-time PCR. For normalization of the PCR results, β-actin (*Actb*) was utilized. The primer sequences of *Bdnf*, *TrkB*, *Akt1*, *Creb1*, and *Actb* are as follow: *Bdnf* (NM_007540), 3'-TCATACTTCGGTTGCATGAAGG-5' and 3'-AGACCTCTCGAACCTGCCC-5'; *TrkB* (NM_001025074), 3'-CTGGGGCTTATGCCTGCTG-5' and 3'-AGGCTCAGTACACCAAATCCTA-5'; *Akt1* (NM_001165894), 3'-ATGAACGACGTAGCCATTGTG-5' and 3'-TTGTAGCCAATAAAGGTGCCAT-5'; *Creb1* (NM_009952), 3'-AGCAGCTCATGCAACATCATC-5' and 3'-AGTCCTTACAGGAAGACTGAACT-5'; and *Actb* (NM_007393), 3'-GGCTGTATTCCCCTCCATCG-5' and 3'-CCAGTTGGTAACAATGCCATGT-5'. The StepOne Real-Time PCR System (Applied Biosystems, Waltham, MA, USA) was used for quantitative PCR (qPCR), and then the results were normalized using those of the control genes encoding β-actin (Actb).

### Western blotting

Proteins were isolated from the hippocampus using RIPA buffer (Sigma, Saint Louis, MO, USA) with PhosSTOP (Roche, Mannheim, Germany) and cOmplete Mini EDTA-free Protease Inhibitor Cocktail (Roche, Mannheim, Germany) supplement on ice. The homogenates were centrifuged at 16,000×*g* for 20 min, and the supernatant was collected. The proteins were normalized by the Quick Start Bradford Protein Assay kit (Bio-Rad, Hercules, CA, USA) following the manufacturer’s instructions. 20 μg of proteins were separated using Mini-PROTEAN TGX Gels, 12% (Bio-Rad, Hercules, CA, USA) then blotted onto Immobilon^®^-P PVDF Membrane (Merck, Darmstadt, Germany). The membranes were blocked for 1 h at room temperature by 5% (w/v) skim milk (BioShop, Burlington, Ontario, Canada) with TBS buffer containing 0.5% (v/v) Tween 20 (Sigma, Saint Louis, MO, USA). The membranes were incubated with primary antibodies against BDNF (1:3,000 dilutions, ab226843, Abcam, Cambridge, UK) or β-actin (1: 5,000 dilutions, 3700S, Cell Signaling Technology, Danvers, MA, USA) for 1 h in room temperature. Subsequent secondary antibody incubation for the membrane was conducted for 1 h at room temperature with horseradish peroxide-conjugated secondary antibodies at 1:3,000 dilution (ADI-SAB-100-J, Enzo Life Sciences, Farmingdale, NY, USA or ab97051, Abcam, Cambridge, UK). The signals were visualized using ECL (Amersham, Little Chalfont, UK) and ImageQuant LAS 4000 (GE Healthcare, Chicago, IL, USA). The signal intensities were quantified using Image J (National Institutes of Health, Bethesda, MD, USA).

### Statistical analysis

Statistical analysis and plot generation were conducted using R (R Core Team, 2021) with the “tidyverse”, “rstatix”, and “ggpubr” packages. The experimental values were visualized as mean ± standard error of the mean (S.E.M.) and differences among groups were evaluated with one-way ANOVA by Tukey’s post hoc test. P-values less than 0.05 were considered significant.

## Results

### Identification of suffruticosol A from seeds of *P. lactiflora*

Specific features were observed in these compounds. The basis of the structure elucidation process was formed by analyzing the ^1^H- and ^13^C-NMR spectra of these compounds. The ^1^H-NMR spectra revealed the presence of six sets of ortho-coupled aromatic hydrogens applied to three 4-hydroxyphenyl groups (A ring of a compound) and signals from three other 3,5-dihydroxy phenyl systems (B ring of a compound) distinctive for three resveratrol units. Instead of the signals for olefinic protons (cis or trans) of resveratrol units, the reduction of these olefinic bonds was recommended by the presence of six methine hydrogens strongly and their trimerization involving these carbons of the three resveratrol units. The analysis of ^13^C-NMR 24 in these compounds gave signals for six phenyl ring systems, including nine oxygenated aromatic quaternary carbons (δc 150.0–160.0), a very deshielded oxymethine (δc 84.0–90.0) and five methine carbons (δc 35.0–66.0). These results supported the hypothesis that these compounds are characterized as resveratrol trimers. In the spectrum results, it showed that the presence of the highly deshielded oxymethine (δ_H_ ~ 6.00, δc ~ 90.0) of each of these three trimers revealed a dihydrofuran ring system. Interestingly, some distinct differences in ^1^H- and ^13^C-NMR signals of these three trimers were observed and this difference suggested that these structures of trimers are significantly different. Thus, based on those data and in comparison with literature^19^, the isolated compound was identified as suffruticosol A in Fig. 1B, C.

### Suffruticosol A elevated viability and cell survival against scopolamine treatments in vitro

**Figure 2:**
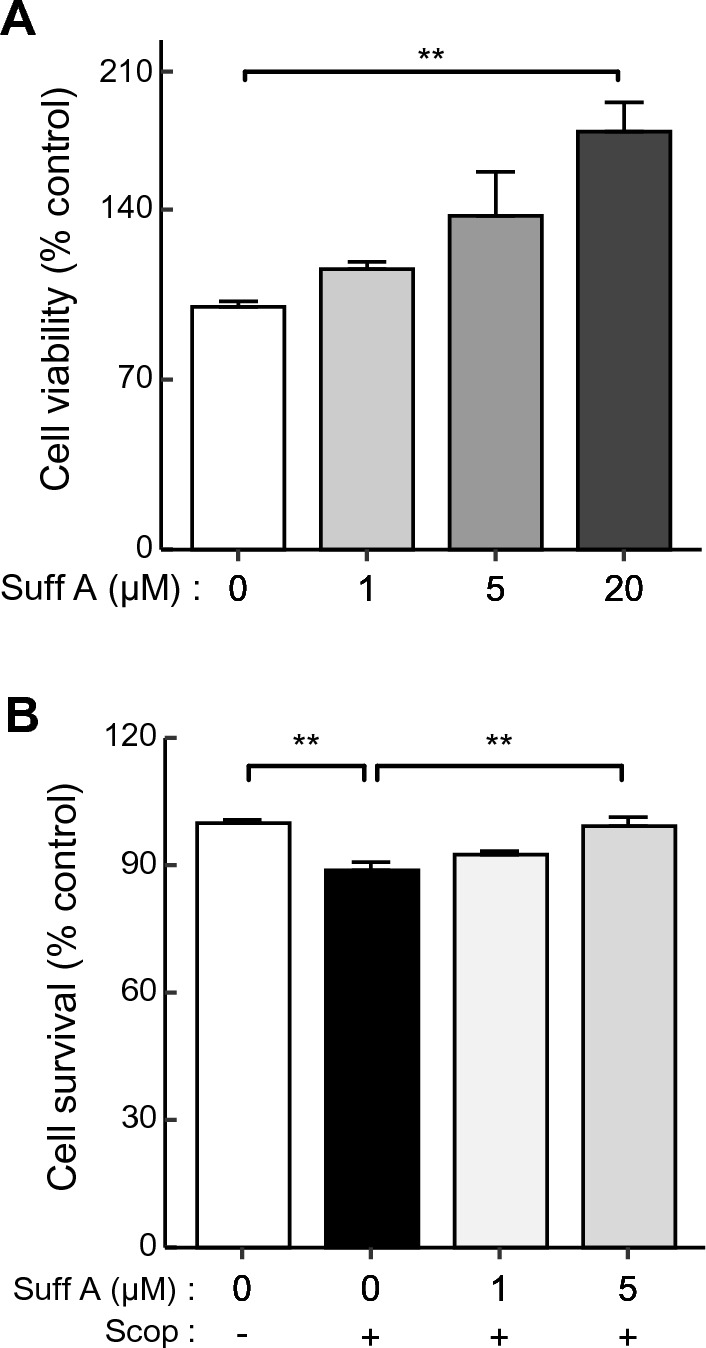
Elevated viability and survival of suffruticosol A on human neuroblastoma SH-SY5Y cells against scopolamine treatment. (**A**) Cells were treated with suffruticosol A (Suff A; 1, 5, or 20 μM) for 24 h and then relative cell viability (%) compared to the control group (0 μM, without scopolamine treatment) was determined by MTT assay. (**B**) Cell survival by treatments of suffruticosol A against scopolamine (Scop; 0 or 2 mM). Values are expressed as mean ± SEM (n = 4). **p < 0.01; one-way ANOVA with Tukey’s post hoc test.

Previous studies have reported therapeutic possibilities of resveratrol on neural activities in vitro^24^, but with insufficient results on its therapeutic effects^26^. Recent research has highlighted the potential of suffruticosol A, a resveratrol trimer, in exerting therapeutic effects against neuronal cell death^27^, which is associated with AD^28^, although evidence is limited. In this study, we utilized a human neuroblastoma cell line, SH-SY5Y, to evaluate the neural survival effects of suffruticosol A in the presence of scopolamine treatment. Prior to assessing the cell survival effects of this compound, we examined its impact on cell viability in SH-SY5Y cells. Treatments with suffruticosol A resulted in a dose-dependent increase in cell viability, with a significant increase observed at 20 μM (Fig. 2A, F_(3,12)_ = 8.081, control vs. Suff A. 20 μM, p < 0.01). Based on these findings, we culture SH-SY5Y cell lines with 0, 1, and 5 μM suffruticosol A in the presence of scopolamine to evaluate its effects on cell survival. While a single treatment of scopolamine (2 mM) reduced cell viability, co-treatment with 5 μM suffruticosol A significantly improved cell (Fig. 2B, F_(3,12)_ = 12.101, control vs. Suff A 0 μM + Scop, p < 0.01, Suff A 0 μM + Scop. vs. Suff A 5 μM + Scop, p < 0.01). These results indicate that suffruticosol A enhances neural activity on the SH-SY5Y cell lines.

### The central administration of suffruticosol A improved memory and cognitive behaviors

**Figure 3:**
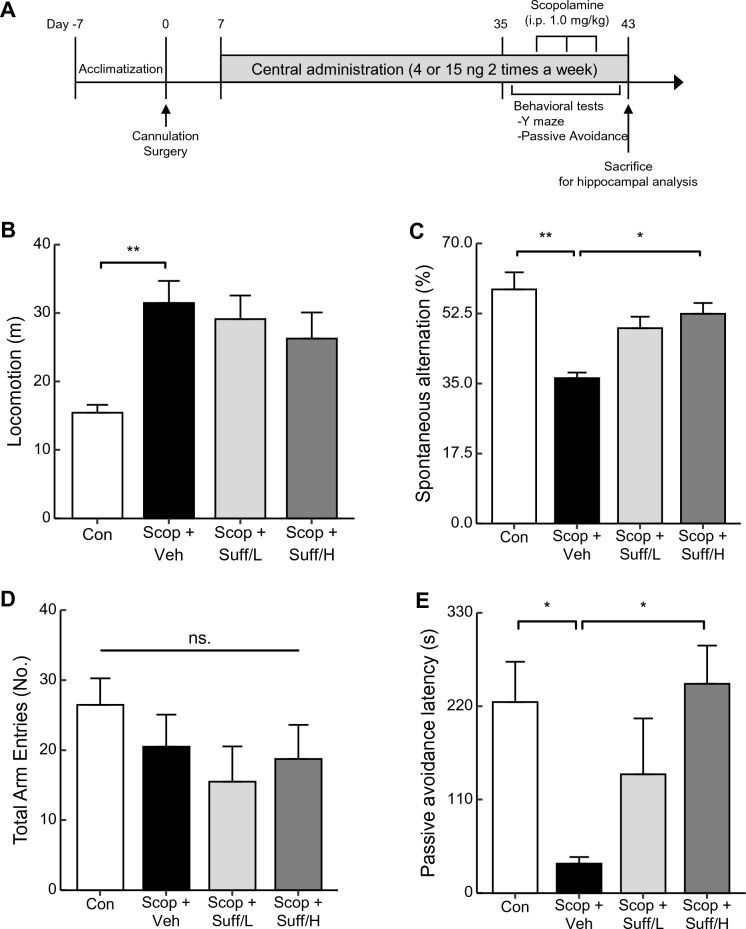
Improved cognition and memory behaviors through central administration of suffruticosol A. Male C57BL/6 mice (8 weeks old) were injected with suffruticosol A (Suff, 4 or 15 ng) or vehicle (Veh, PBS) into the third-ventricle via cannula twice a week for 1 month. (**A**) The schematic experimental schedules. Before the cannulation surgery, all mice were acclimated for 1 week. The mice were rehabilitated for another week and then central administration of suffruticosol A or vehicle was conducted for 1 month. (**B–D**) After the administration, behavioral tests were performed. Scopolamine (Scop, 1.0 mg/kg, i.p.) or 0.9% saline was injected 30 min before each behavioral test. The locomotion in open field (**B**), spontaneous alternation (**C**) and total arm entries (**D**) in Y-maze, and passive avoidance (**E**) were conducted on control (Con, PBS as a vehicle), scopolamine + vehicle (Scop + Veh), scopolamine + suffruticosol 4 ng (Scop + Suff/L), and scopolamine + suffruticosol 15 ng (Scop + Suff/H). Data are expressed as mean ± SEM (n = 5). *p < 0.05, **p < 0.01; one-way ANOVA with Tukey’s post hoc test.

Building upon the elevated neural activities observed with suffruticosol A in vitro, we aimed to investigate whether treatment with suffruticosol A via the third ventricle of the brain could enhance memory and cognition in experimental animals that received scopolamine to induce deficits in these functions. For accurate experimental procedures, we designed the schedules as depicted in Fig. 3A. Subsequently, stereotaxic surgery was performed to implant the cannula into the third ventricle of the brain. To determine the appropriate dose of suffruticosol A for central administration, we reviewed and analyzed several studies using central administration^37–40^. Based on these analyses, we modified the formula which we used in our previous studies for selecting the optimal dose for the brain^33,41^. Then, we applied the results of cell lines (from Fig. 2) to this formula for deciding the dose of central administration in animals^42^. Firstly, we converted the units from μM to ng/μl, rounded them to whole numbers, and then multiplied them by two, as demonstrated in Eq. (1).

1 $$\mathrm{dose }\left(\frac{\mathrm{ng}}{{\upmu {\rm l}}}\right)=\left[\mathrm{concentration }\left({\upmu {\rm M}}\right)\times \mathrm{molecular\, weight }\left(\frac{\mathrm{g}}{\mathrm{mol}}\right)\right]\times 2$$

Consequently, we administered a low dose of suffruticol A (Suff/L, 4 ng) or a high dose of suffruticosol A (Suff/H, 15 ng) through a cannula into the brains of the animals. Initially, we did central administration for 3 days for expecting the acute effect of this chemical. However, the results of Y-maze tests showed no significant changes by suffruticosol A treatment (data not shown). In this study, the central treatments of suffruticosol A were relatively low doses (4 ng or 15 ng), compared to those in other reference use (µg or mg)^37,39,40^. Thus, these low-dose treatments are suitable for inducing the chronic effects of suffruticosol A. To evaluate the long-term effect of suffruticosol A, we kept the frequency of central administration twice a week for one month. Following the chronic treatments of suffruticosol A (1 month), scopolamine (1.0 mg/kg) was intraperitoneally administered to the Veh, Suff/L, and Suff/H groups prior to the behavioral tests (30 min in advance). The scopolamine injection in mice leads to increased locomotion^43^. To confirm the proper action of scopolamine, we measured the locomotion of these experimental mice by open-field tests. The treatments of scopolamine induced significantly higher activities in the mice but treatments of suffruticosol A affected trends in increased locomotion without significance against the treatments of scopolamine (Fig. 3B, F_(3,16)_ = 8.219, Con vs. Scop + Veh, p < 0.01). To measure short-term memory, we tested a Y-maze test for analyzing spontaneous alternation. This alternation was inhibited by scopolamine treatments but protected in the high dose of suffruticosol A (Fig. 3C, F_(3,12)_ = 9.776, Scop + Veh vs. Scop + Suff/H, p < 0.05). The total arm entries of all groups showed no differences (Fig. 3D, F_(3,12)_ = 1.011, One-way Anova, p = 0.422) which refers lowered spontaneous alternation of Scop group is not derived from hyperactivity but from memory impairment. To examine whether the treatments of suffruticosol A had long-term effects on memory, we conducted passive avoidance tests on these mice. Consistently, scopolamine treatments impaired memory, while high doses of suffruticosol A induced the restoration of memory (Fig. 3E, F_(3,19)_ = 4.838, Con vs. Scop + Veh, p < 0.05, Scop + Veh vs. Scop + Suff/H, p < 0.05). This suggests that the chronic treatments of suffruticosol A rescue neural activity against memory deficit induced by the scopolamine treatments.

### Suffruticosol A restored the impairments of the cholinergic systems by the scopolamine treatments in the hippocampus

**Figure 4:**
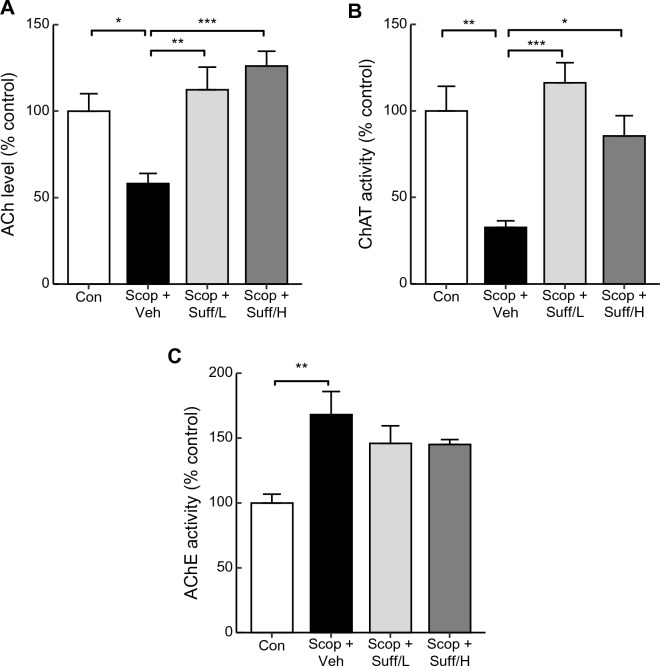
Effect of suffruticosol A on hippocampal cholinergic function. Male C57BL/6 mice (8 weeks old) were injected with suffruticosol A (4 or 15 ng) or vehicle (PBS for control group) into the third-ventricle via cannula twice a week for one month. The mice were sacrificed and hippocampi were collected for measurement of cholinergic functions. 30 min before the sacrifice, scopolamine (1.0 mg/kg, i.p.) was injected to each group; scopolamine + vehicle (Scop + Veh), scopolamine + suffruticosol 4 ng (Scop + Suff/L), and scopolamine + suffruticosol 15 ng (Scop + Suff/H). The control group (Con) was injected with 0.9% saline (i.p.). (**A–C**) The hippocampal acetylcholine (ACh) level, choline acetyltransferase (ChAT) activity, and acetylcholine esterase (AChE) activity were measured (n = 5). Data are expressed as mean ± SEM (n = 5). *p < 0.05, **p < 0.01, ***p < 0.001; one-way ANOVA with Tukey’s post hoc test.

Depletion of the cholinergic system leads to a reduced BDNF signaling, and these factors are associated with cognitive deficits^44^. To determine whether suffruticosol A treatments in the brain can restore cholinergic system activity, we assessed the activities of choline acetyltransferase (ChAT), acetylcholine contents (ACh), and acetylcholinesterase (AChE), which are crucial for neuronal functions. The treatments of scopolamine significantly decreased ACh levels and ChAT activities (Fig. 4A, B, A: F_(3,16)_ = 8.986, Scop + Veh vs. Scop + Suff/L, p < 0.01, Scop + Veh vs. Scop + Suff/H, p < 0.001, B: F_(3,16)_ = 10.671, Scop + Veh vs. Scop + Suff/L, p < 0.001, Scop + Veh vs. Scop + Suff/H, p < 0.05). However, the treatments of suffruticosol A restored ACh levels and ChAT activities in both the Scop + Suff/L and Scop + Suff/H groups (Fig. 4A, B). In contrast, scopolamine treatments significantly increased AChE activity, but suffruticosol A treatments did not restore this activity (Fig. 4C, F_(3,16)_ = 5.832).

### Central administration of suffruticosol A elevates BDNF signaling

**Figure 5:**
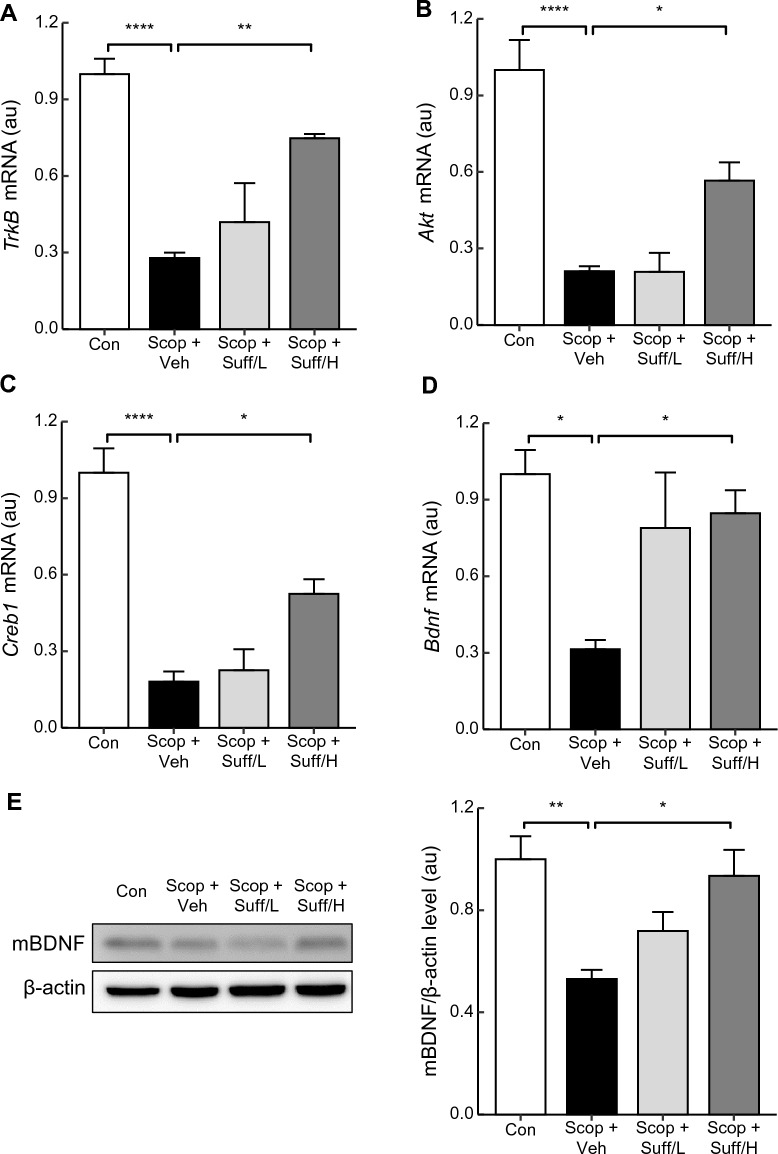
Increased BDNF signaling by central administration of suffruticosol A. Male C57BL/6 mice (8 weeks old) were injected with suffruticosol A (4 or 15 ng) or vehicle (PBS) into the third-ventricle via cannula twice a week for one month. The mice were sacrificed and hippocampi were collected for measurement of mRNA expression. 30 min before the sacrifice, scopolamine (1.0 mg/kg, i.p.) was injected to each group; scopolamine + vehicle (Scop + Veh, PBS as vehicle), scopolamine + suffruticosol 4 ng (Scop + Suff/L), and scopolamine + suffruticosol 15 ng (Scop + Suff/H). The control group (Con) was treated with vehicle (PBS) and injected with 0.9% saline (i.p.). (**A–D**) mRNA levels related to BDNF signals were measured in the hippocampus (n = 4–5). The mRNA expression was normalized to that of β-actin. Au means arbitrary units. (**E**) The relative intensity of hippocampal mBDNF protein was measured by western blotting and normalized to that of β-actin (n = 4–5). The mBDNF protein levels were then divided by the mean value of the control group. The original blots are presented in Supplementary Fig. 1. Data are expressed as mean ± SEM (n = 4–5). *p < 0.05, **p < 0.01, ****p < 0.0001; one-way ANOVA with Tukey’s post hoc test.

To further elucidate the molecular mechanism underlying the restoration of neural activity, we analyzed the mRNA expression of *Bdnf, TrkB, Akt,* and *Creb1*. These signaling pathways are closely associated with cognition and memory function, and the treatments of scopolamine down-regulate BDNF signaling^10^. Mature BDNF (mBDNF) binds to the TrkB receptor, activating Akt and cAMP response element-binding protein (CREB) signaling, which enhances neuronal survival, growth, and synaptic plasticity regulation^45,46^. Therefore, Suffruticosol A treatments may restore the impaired BDNF signaling caused by scopolamine treatments. We extracted RNAs from the hippocampi to analyze the mRNA expression of the BDNF signaling cascades. Interestingly, the high-dose Suffruticosol A treatments significantly restored BDNF signaling that was impaired by scopolamine (Fig. 5A–C). The mRNA expression of *TrkB*, *Akt*, and *Creb1*, which were down-regulated by scopolamine treatment, were rescued significantly in the Scop + Suff/H groups (5A: F_(3,14)_ = 19.631, Scop + Veh vs. Scop + Suff/H, p < 0.01, 5B: F_(3,14)_ = 22.845, Scop + Veh vs. Scop + Suff/H, p < 0.05, 5C: F_(3,14)_ = 28.666, Scop + Veh vs. Scop + Suff/H, p < 0.05). The mRNA expression of BDNF was significantly restored in the Scop + Suff/H groups (Fig. 5D, F_(3,13)_ = 5.398, Scop + Veh vs. Scop + Suff/H). The TrkB receptor shows a strong affinity to the mature form of BDNF^45^. To examine the effect of suffrutiocosol A on mBDNF restoration, we conducted western blotting to mBDNF expression. The protein level of mBDNF was decreased in Scop + Veh groups while significantly recovered in Scop + Suff/H groups (Fig. 5E, F_(3,14)_ = 6.504, p < 0.05). These findings indicate that suffruticosol A ameliorates mBDNF levels against scopolamine treatment.

### Suffruticosol A protects the scopolamine-induced impairment of long-term potentiation in the hippocampus

**Figure 6:**
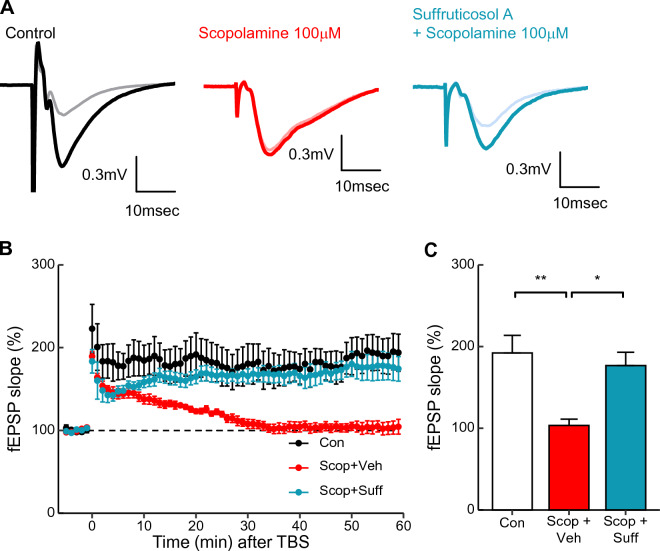
Protective effect of suffruticosol A against scopolamine-induced impairment of hippocampal LTP. (**A**) Representative traces for averaged evoked EPSP of baseline for 5 min (gray, pink, sky blue) and last 5 min after TBS stimulation (black, red, cyan) in control, 100 µM scopolamine with DMSO vehicle and 100 µM scopolamine with 40 µM suffruticosol A, respectively (scale bars, 0.3 mV, 10 ms). (**B**) Averaged traces of normalized percentage of fEPSP slope in control (black), 100 µM scopolamine (red), and 40 µM suffruticosol A with 100 µM scopolamine (cyan). (**C**) Averaged bar graph for normalized percentage of fEPSP recorded last 5 min. Values are expressed as means ± SEM (n = 6 for control and scopolamine, n = 7 for suffruticosol A with scopolamine; * *p* < 0.05, ** *p* < 0.01; one-way ANOVA with Tukey’s multiple comparisons test).

Long-term potentiation (LTP), a long-lasting enhancement of synaptic transmission, is supposed to be the type of synaptic plasticity that motivates hippocampal learning and memory functions^47^. We investigated whether the bath application of suffruticosol A could restore the LTP impairment induced by the scopolamine in the hippocampus. We applied a single dose of suffruticosol A (40 μM) to the slices of hippocampal tissues, which were perfused with artificial cerebrospinal fluid (aCSF) containing either DMSO vehicle or scopolamine (100 μM). In the control group, the field excitatory postsynaptic potential (fEPSP) was significantly increased and maintained for 60 min after TBS stimulation (Fig. 6). In the scopolamine-treated group (Scop + Veh), LTP was completely impaired (Fig. 6C, F_(2,16)_ = 8.229, p < 0.001). Remarkably, the combined treatment of suffruticosol A restored the scopolamine-induced LTP impairment (Fig. 6C, p < 0.05). These findings suggested that suffruticosol A protects against the scopolamine-induced impairment of LTP in the hippocampus. This effect could be attributed to suffruticosol A acting as a synaptic mechanism that contributes to the neuroprotective properties of suffruticosol A in hippocampal memory.

## Discussion

*Paeonia lactiflora* is a medicinal herb commonly used in traditional Chinese medicine. This herb has been known to prevent blood clotting, carcinogenesis, and inflammation partly due to its strong antioxidant capacity^48,49^. In contrast, the seeds of this herd gained less attention for medicinal purposes. In this study, we found that the seeds of *P. lactiflora* have a complex structure of resveratrol, called suffruticosol A, and this chemical showed ameliorative effects against scopolamine-induced dementia. Resveratrol is an intriguing chemical because it has a polyphenolic antioxidant and can be easily found in many plants, such as grapes, berries, and nuts^50^. Since its structure was identified in 1940 from the roots of white hellebore, it has gained eminence associated with the ‘French Paradox’ related to the consumption of red wine. Consuming red wines refers to the very low incidence of and mortality rates from cardiovascular diseases in the French^51^. Recently, resveratrol derivatives from grapes, *Vitis vinifera*, also have shown strong neuronal amelioration activities in dementia^52^. In this study, we found an interesting derivative structure of resveratrol isolated from the seeds of *P. lactiflora.* Suffruticosol A abundant in the seeds of *P. lactiflora* may be used as a therapeutic agent against dementia partially due to its neuronal amelioration effects.

In AD patients, cholinergic impairments usually occur in the hippocampus, nucleus basalis of Meynert, and cortex^53^, which contributes to memory deficits^54^. An animal model of scopolamine-induced memory deficits, similar to the AD^9^. Scopolamine is a non-selective antagonist of a muscarinic receptor (M receptor) that blocks a neurotransmitter, acetylcholine (ACh)^55^. The M1 receptors are predominant in the cortex and hippocampus^56^. Blocking of M1 receptors causes damage to the hippocampus through the excessive release of ACh^57^, and reduces the long-term potentiation (LTP)^58^. The impaired hippocampal LTP is related to cognitive deficit because of its role in the learning and memory process^58–60^. Thus, scopolamine treatment induces a decrease in the cholinergic system and a loss of memory which resembles the AD patients^9,61^. Furthermore, scopolamine treatments do not necessitate complex surgical procedures, making this chemical a common choice for investigating therapeutic agents for neurodegenerative diseases.

The scopolamine-induced memory deficit model exhibits similarities in the downregulated brain-derived neurotrophic factor (BDNF) levels in AD patients^62^. BDNF signaling has a crucial role in AD, as evidenced by the low mRNA and protein expression levels of BDNF observed in AD patients^63,64^. Post-mortem examination of brains from AD patients revealed a loss of BDNF expression in both reactive microglia and neurons, which contained massive neurofibrillary tangles compared to normal neurons^65^. Prolonged depletion of ACh in the nucleus basalis downregulates ACh muscarinic receptor activation^44^. This downregulation depletes BDNF mRNA transcription and mature BDNF (mBDNF) protein level^44^. Scopolamine treatment hinders non-selective muscarinic receptor activation which induces a reduction of BDNF levels. Furthermore, scopolamine reduces the expression level of the mBDNF receptor, TrkB^66^. Restoring cholinergic function could re-establish mBDNF level. The mRNA level of related signals such as *Akt* and *Creb1* may indicate the increase of mBDNF is derived from the cAMP response elements-binding protein (CREB) signaling cascade, with limited evidence. Based on our study, the administration of suffruticosol A postponed the deficit of cholinergic function and impairment of cognition and memory. The administration demonstrated improvements in long-term memory, partially attributed to the restoration of cholinergic function and BDNF signaling.

The cholinergic functions induce neurogenesis in the hippocampus via the BDNF signaling pathway, which is necessary for long-term potentiation (LTP)-memory formation. LTP refers to a long-lasting increase in the effectiveness of excitatory synaptic transmission. Hippocampal LTP is commonly responsible for a cellular signaling cascade of learning and memory^59,60^. BDNF and glutamate are mostly working on memory functions^67^. BDNF is tightly related to the LTP by directly working on depolarizing neurons by increasing glutamatergic transmission for inducing phosphorylation of NMDA signaling via its TrkB receptors^68^. The BDNF positively controls LTP, promoting memory formation at the cellular and molecular levels. In addition, treatments of recombinant BDNF rescued impaired LTP in BDNF-mutant mice^69^. The LTP formation, which is connected to presynaptic neurons, depends on BDNF protein synthesis^69,70^. Thus, the BDNF regulates the translation of protein synthesis through several intracellular signaling pathways, related to cell growth, survival, differentiation, and intracellular trafficking via Akt and PI3K signaling. Our study presented that the central administration of suffruticosol A into the brain restores the deficits, impaired by scopolamine treatments, in hippocampal LTP partly through inducing BDNF activation. These recovered activities and the enhanced capability of LTP in the brain might contribute to neuroprotection in neurodegenerative diseases.

## Conclusion

The treatments of suffruticosol A enhanced neural activity in cell lines, restored LTP-memory formation, recovered the cholinergic system, and increased memory and cognitive behaviors through partly BDNF signaling. These findings suggest that suffruticosol A might be considered a therapy for neurodegenerative disease.

## Supplementary Information

Supplementary Figure 1.

## Data Availability

All data sources of this study are spawned in the article.
