# Supplementary material for: Enhanced neuronal activity by suffruticosol A extracted from Paeonia lactiflora via partly BDNF signaling in scopolamine-induced memory-impaired mice
Source: Sci Rep. 2023 Jul 20;13:11731. doi: 10.1038/s41598-023-38773-8 (PMC10359324; doi:10.1038/s41598-023-38773-8)
Supplement: Supplementary file 1 — Supplementary Figure 1. [file 41598_2023_38773_MOESM1_ESM.pdf]

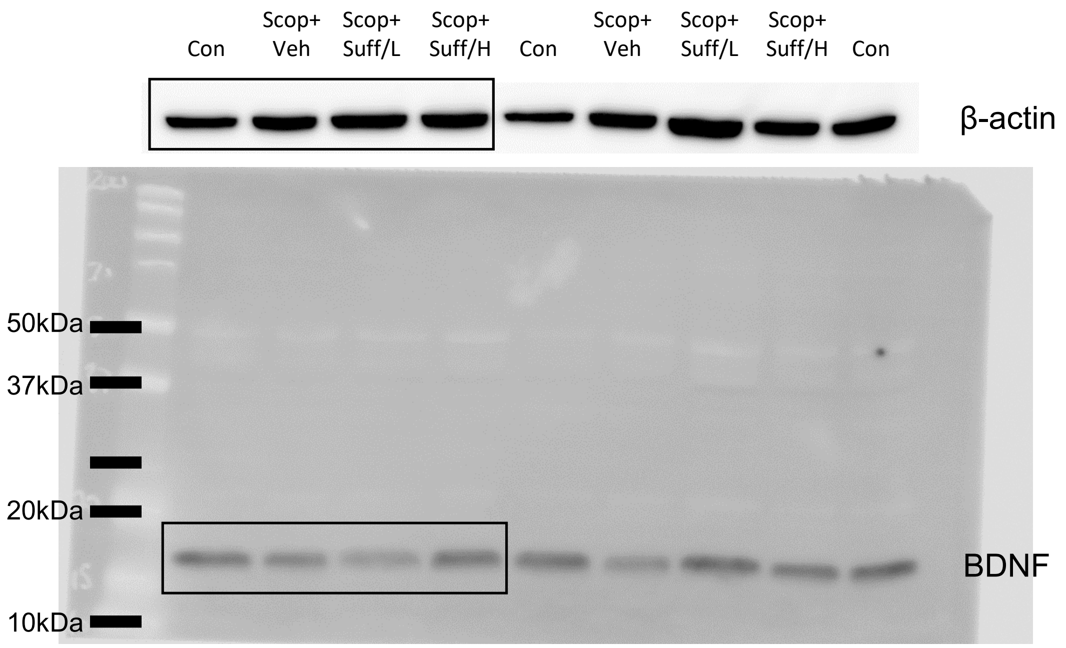

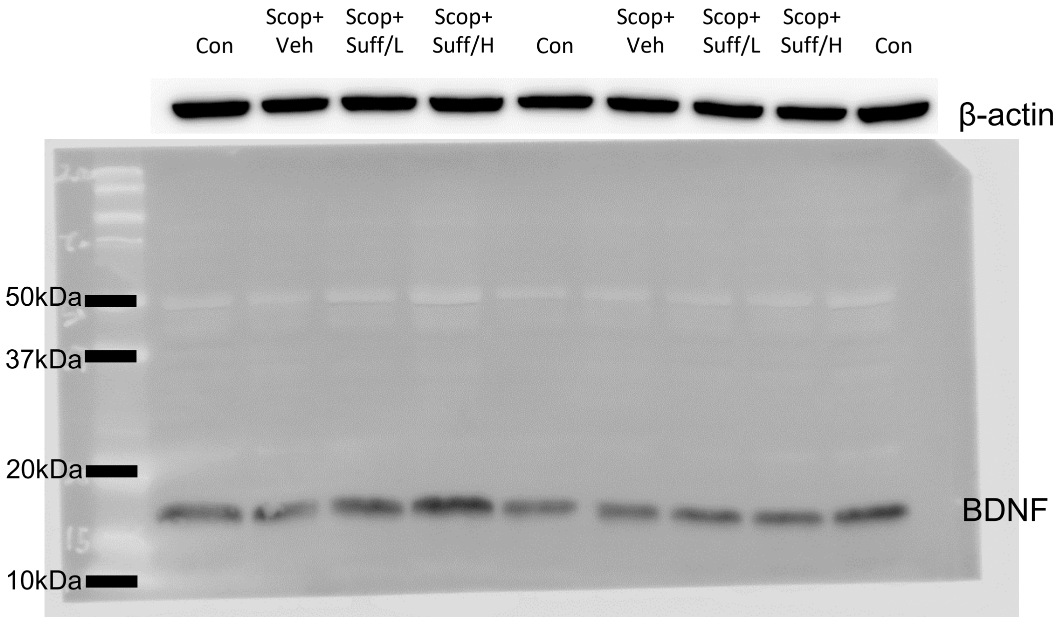

Supplementary Figure 1. The images of full-length blots of BDNF and β-actin proteins in hippocampus.

Male C57BL/6 mice (8 weeks old) were injected with suffruticosol A (4 or 15 ng) or vehicle (PBS) into the third-ventricle via cannula twice a week for one month. The mice were sacrificed and hippocampi were collected. 30 min before the sacrifice, scopolamine (1.0 mg/kg, i.p.) was injected to each group; scopolamine + vehicle (Scop+Veh, PBS as vehicle), scopolamine + suffruticosol 4 ng (Scop + Suff/L), and scopolamine + suffruticosol 15 ng (Scop+Suff/H). The control group (Con) was treated with vehicle (PBS) and injected PBS (i.p.). The samples derived from the same experiment and that gels/blots were processed in parallel and these images were used for the representative blot pictures in Fig. 5E
